# Progress towards reducing sociodemographic disparities in breastfeeding outcomes in Indonesia: a trend analysis from 2002 to 2017

**DOI:** 10.1186/s12889-020-09194-3

**Published:** 2020-07-15

**Authors:** Nurmala Selly Saputri, Belinda Rina Marie Spagnoletti, Alison Morgan, Siswanto Agus Wilopo, Ankur Singh, Barbara McPake, Rifat Atun, Rika Kumala Dewi, John Tayu Lee

**Affiliations:** 1 Melbourne School of Population and Global Health, University of Melbourne, Melbourne, Australia; 2 The SMERU Research Institute, Central Jakarta, Indonesia; 3 Nossal Institute for Global Health, Melbourne School of Population and Global Health, University of Melbourne, Melbourne, Australia; 4 Center for Reproductive Health, Faculty of Medicine, Nursing and Public Health, Gadjah Mada University, Yogyakarta, Indonesia; 5 Biostatistics, Epidemiology, and Population Health, Faculty of Medicine, Public Health and Nursing, Gadjah Mada University, Yogyakarta, Indonesia; 6 Centre for Health Equity, Melbourne School of Population and Global Health, University of Melbourne, Melbourne, Australia; 7 Harvard T.H Chan, School of Public Health, and Harvard Medical School, Harvard University, Cambridge, USA; 8 School of Primary Care and Public Health, Imperial College London, London, England

**Keywords:** Breastfeeding, Indonesia, Maternity protection, Time trend analysis

## Abstract

**Background:**

Improving breastfeeding practice is important for reducing child health inequalities and achieving several Sustainable Development Goals. Indonesia has enacted legislation to promote optimal breastfeeding practices in recent years. We examined breastfeeding practices among Indonesian women from 2002 to 2017, comparing trends within and across sociodemographic subgroups.

**Methods:**

Data from four waves of the Indonesia Demographic and Health Surveys were used to estimate changes in breastfeeding practices among women from selected sociodemographic groups over time. We examined three breastfeeding outcomes: (1) early initiation of breastfeeding; (2) exclusive breastfeeding; and (3) continued breastfeeding at 1 year. Multivariate logistic regression was used to assess changes in time trends of each outcome across population groups.

**Results:**

The proportion of women reporting early initiation of breastfeeding and exclusive breastfeeding increased significantly between 2002 to 2017 (*p* < 0.05), with larger increases among women who: were from higher wealth quintiles; worked in professional sectors; and lived in Java and Bali. However, 42.7% of women reported not undertaking early initiation of breastfeeding, and 48.9% of women reported not undertaking exclusive breastfeeding in 2017. Women who were employees had lower exclusive breastfeeding prevalence, compared to unemployed or self-employed women. Women in Java and Bali had higher increase in early initiation of breastfeeding and exclusive breastfeeding compared to women in Sumatra. We did not find statistically significant decline in continued breastfeeding at 1 year over time for the overall population, except among women who: were from the second poorest wealth quintile; lived in rural areas; did not have a health facility birth; and lived in Kalimantan and Sulawesi (*p* < 0.05).

**Conclusions:**

There were considerable improvements in breastfeeding practices in Indonesia during a period of sustained policy reform to regulate breastfeeding and community support of breastfeeding, but these were not distributed uniformly across socioeconomic, occupation and geographic subgroups. Concerted efforts are needed to further reduce inequities in breastfeeding practice through both targeted and population-based strategies.

## Background

In 2015 the United Nations Annual Meeting on Breastfeeding stressed the importance of breastfeeding to reduce child mortality [1]. Breastfeeding is important for achieving several Sustainable Development Goals (SDGs), specifically SDG 3, concerned with promoting health and well-being, and SDG 2, concerned with ending hunger and achieving food security [2, 3]. The World Health Organization (WHO) recommends early initiation of breastfeeding (EIB), exclusive breastfeeding (EB) and continued breastfeeding up to 2 years of age as optimal breastfeeding practices [4]. EIB refers to infants being breastfed within one hour after birth, while EB is when infants only receive breast milk for the first 6 months of life [5, 6]. Breastfeeding, especially EB, also helps to protect children from infection, reduce the prevalence of asthma and obesity, and increase children’s cognitive ability [7–9]. Non-exclusive breastfeeding caused 1.4 million deaths and 10% of disease burden in children aged under five [10]. The benefits of breastfeeding for women include reduced risk of breast and ovarian cancers and type 2 diabetes, and improved birth spacing [7].

In Indonesia, the infant mortality rate was 21.4 per 1000 live births in 2017, higher than other lower- and middle-income countries (LMIC) in Southeast Asia, including Vietnam (17 per 1000 live births), Thailand (8 per 1000 live births), and Malaysia (7 per 1000 live births) [11]. According to the 2018 Indonesian Basic Health Survey (*Riskesdas*), EB and EIB rates in Indonesia were only 37.3 and 58.2%, respectively [12]. Bangka Belitung province had the highest rate of EB (56.7%), while West Nusa Tenggara province had the lowest prevalence (20.3%). Several recent studies have identified barriers to optimal breastfeeding practices in Indonesia, including: limited knowledge of breastfeeding among mothers and health workers; promotion of infant formula in retail outlets and by health workers; limited duration of maternity leave postpartum; and logistical challenges for women who return to the workforce after maternity leave [13–18].

Since 2003, the Government of Indonesia (GoI) has introduced several laws and policies to encourage greater breastfeeding prevalence among Indonesian women. The 2003 Labor Law obliges employers to provide three-months paid maternity leave and breastfeeding breaks during working hours. The National Health Law 36/2009 stipulates the right of every child to be exclusively breastfed. In 2008, a Joint Regulation issued by the Ministry of Women’s Empowerment, the Ministry of Manpower and Transmigration, and the Ministry of Health, emphasized the importance of breastfeeding promotion and ensuring the provision of lactation rooms and breastfeeding breaks for working women [19]. Government Regulation 33/2012 on Exclusive Breastfeeding, introduced in 2012, recommended community-level promotion of EB. Regulation 33/2012 complements prior legislation, providing a detailed explanation of ideal provisions for lactation rooms in workplaces, and the prohibition of infant formula promotion [20]. Provincial and district governments are encouraged to adopt Regulation 33/2012, although the lacking implementation of this legislation has been noted by Spagnoletti et al. [15]. Aside from these pieces of legislation, the GoI has a stated target to achieve minimum rates of 50% for EIB and EB in the National Medium-Term Development Plan 2015–2019 and the Strategic Plan of the Ministry of Health 2015–2019 [21, 22].

In 2017, the WHO’s Global Breastfeeding Scorecard evaluated breastfeeding promotion policies and interventions globally [23]. There are at least eight indicators used to determine the level of commitment of each country in protecting breastfeeding, including the implementation of legislation in alignment with the International Code of Marketing of Breast-Milk Substitutes (the Code) and the Baby-Friendly Hospital Initiative (BFHI). Although Indonesia has adopted the Code and BFHI since 2012 and 1994 respectively, both interventions have been poorly implemented. For example, excessive promotion of infant formula is still commonplace in Indonesia [24]. The regulation of infant formula promotion in Indonesia does not include provisions pertaining to community-based workers who play an important role in promoting breastfeeding [24]. Thus, no fines nor sanctions can be given to infant formula companies that collaborate with community-based workers. Moreover, Hidayana et al. [25] reported that some health workers received gifts from infant formula companies, infant formula labelling contained content that discouraged breastfeeding practice, and free formula samples were being distributed to mothers. In the case of the BFHI, in 2015 just 8% of hospitals in Indonesia had been designated as BFHI [26].

While earlier studies have highlighted some of the factors associated with breastfeeding practices in Indonesia [27–29], this prior research has not explored breastfeeding trends over time. High socioeconomic status, low education status, low knowledge, labour force participation, and complications during birth are found to affect breastfeeding practices [27, 28, 30, 31]. However, there is limited understanding of the relationship between such socioeconomic characteristics and trends in relation to breastfeeding outcomes. This study aimed to examine breastfeeding trends from 2002 to 2017 by: maternal socioeconomic, education and occupation status; place and region of residence; place of delivery; and birth assistance type. The main hypothesis in this study is that the introduction of legislation designed to promote breastfeeding by the GoI has led to improvements in breastfeeding outcomes across subgroups. The secondary hypothesis is that the various legislations to support working mothers to breastfeed has also increased breastfeeding practices among women who are employed.

## Methods

### Data and study sample

This research uses four waves of data from the Indonesia Demographic and Health Survey (IDHS): 2002/2003, 2007, 2012, and 2017. The IDHS data used in this research allow for national and provincial level estimations, as it is representative at both administrative levels.^1^ The IDHS 2002/2003, 2007, 2012 and 2017 collected data from 33,088, 40,701, 43,852, and 47,963 households, respectively [32–35].

The IDHS 2002/2003 and 2007 collected information from ever-married women aged 15–49 years, while the IDHS 2012 and 2017 samples included all women aged 15–49 years. In our analysis, we included ever-married women with a singleton child below 24 months and only included the youngest child. Based on the inclusion criteria, there are total 26,050 observations in the dataset: 5980 women from IDHS 2002/2003; 6898 from IDHS 2007; 6698 from IDHS 2012; and 6474 from IDHS 2017 (see Appendix 1 for sample flow chart).

### Variables

We examined three breastfeeding outcomes: (1) early initiation of breastfeeding (EIB), among children 0–23 months; (2) early breastfeeding (EB) among children aged 0–5 months; and (3) continued breastfeeding at 1 year (CB-1) among children 12–15 months. The population considered in deriving these outcomes were consistent with previous reports published by the WHO, and other international agencies [6].

For the EIB variable, respondents were asked: “How long after birth did you first put (name) to the breast?”. The study defined those who answered ‘immediately, below 1 hour’ as EIB. The study population for this outcome is women with singleton and youngest child aged 0–23 months. The EB variable uses two similar questions. First, the respondents were asked: “Are you still breastfeeding (name)?”. After that, the respondents were asked about the foods and liquids given to their children 24 h before the survey. Those who only gave breastmilk are categorized as exclusively breastfed in the EB variable. The study population for the EB outcome are women with singleton and youngest child aged 0–5 months. The question about the current status of breastfeeding is used to construct the CB-1 variable. The respondents were asked: “Are you still breastfeeding (name)?”. The continued breastfeeding at 1 year is when respondents still gave breastmilk to their children aged 12–15 months.

Seven variables were created to analyze disparities in breastfeeding practices in Indonesia. These variables include: (i) wealth index (poorest, poorer, middle, richer, richest); (ii) education status (no education/incomplete primary, primary/incomplete secondary, post-secondary); (iii) occupation status (unemployed, employee in professional sectors, employee in other sectors, and self-employed/work for family); (iv) place of residence (urban, rural); (v) geographical region (Sumatra, Java & Bali, Kalimantan, Sulawesi, and Eastern Indonesia); (vi) place of delivery (non-health facility, government health facility, private health facility) and (vii) birth assistance (non-skilled birth attendant and skilled birth attendant).

The wealth index was constructed using principal component analysis (PCA) applied to household ownership of selected assets, including drinking water source, electricity access, and toilet type. The details of wealth index construction can be found elsewhere [36]. The ‘professional’ occupation grouping includes women in the technical management, administration and clerical sectors. The occupation grouping described as ‘other’ includes those in the sales, agricultural, industrial and service sectors (for more detail on measurement of variables see Appendix 2).

### Statistical approach

For each breastfeeding outcome, we described prevalence at specific time points and changes in breastfeeding prevalence within each socioeconomic, education, occupation, residence, and regional group. Prevalence was age-standardized, using the age distribution of women, as per the Indonesian Census 2000. We calculated absolute differences for each indicator with a 95% confidence interval. We examined time trends between 2002/2003 to 2017 by fitting a multivariable logistic regression model for each breastfeeding outcome and time as a covariate (set to 0 in years 2002/2003, equal to number of years since 2003). The models were adjusted for maternal sociodemographic characteristics; pregnancy-related factors, including the desire to have children; baby’s size at birth; birth attendant type; mode of delivery; and place of delivery. For examining time trends within subgroups, the above analyses were stratified by socioeconomic status, education, occupation, residence and region. We also compared outcomes in each year with those in 2002/2003 (the reference period) by fitting dummy variables for each survey year and presenting prevalence odds ratios (pORs). Prevalence odds ratio (pORs) is commonly used as a measure of association in cross-sectional studies that include prevalent cases [37]. It has a similar meaning and interpretation with odds ratio (OR), as it is calculated in the same manner. The odds of breastfeeding practices in other years are compared with the year 2002/2003 as the reference period.

To obtain appropriate national estimates to allow comparison across waves, all analyses were weighted to the respondent’s probability of selection and the age, sex-specific population from annually adjusted intercensal estimates. We used SVY command with PSU (primary sampling unit) in STATA 15 to account for the two-stage cluster sampling design. The multicollinearity diagnostic for covariates (variance inflation factor) were all less than ten, indicating the assumption of reasonable independence among predictors was met [38].

## Results

### Sample characteristics

This study analysed data from 26,050 women, who had a child aged 0–23 months at the time of survey completion, from four waves of IDHS. There were less than 5% of missing data for each variable. The overall analysis showed that just over half of the study population was concentrated in Java and Bali (55.86%) and rural Indonesia (53.14%) (see Appendix 3). More than half of the respondents had completed primary school (51.37%) and were unemployed (58.76%). The percentage of women from the lowest wealth quintile was slightly higher (20.6%) than women from other wealth quintile groups.

### Early initiation of breastfeeding

EIB prevalence for 2002/2003, 2007, 2012, and 2017 was 35.90% (95% CI: 32.60–39.19%), 39.41% (95% CI: 36.85–41.96%), 50.55% (95% CI: 48.14–52.95%), and 57.29% (95% CI: 55.14–59.43%), respectively. Table 1 presents the trend in prevalence of EIB for the entire population, as well as within population subgroups. Overall, the pORs shows that EIB increased between 2002/2003 and 2017 for the entire population, as well as within all population subgroups. The increase was greater among women who were from the richest wealth quintile compared to the poorest one, the pOR was 1.56 (95% CI: 1.43–1.7) and 1.29 (95% CI: 1.20–1.39), respectively. However, the increases in EIB were similar for respondents across educational, occupational, and geographical groups. Therefore, the disparity remained in region groups, where women in Sumatra (45.38%) and Sulawesi (46.10%) had lower rates of EIB in 2017, compared to women in other regions.

**Table 1:** Age-standardised proportion of women who had EIB, by select characteristics

**Variable**	**2002/2003**	**2007**	**2012**	**2017**	**Diff. between 2002/2003−2017**	**95% CI**	**pOR**^**a**^	**95% CI**
Overall	35.90	39.41	50.55	57.29	**21.39**	17.45–25.32	**1.43**	1.37–1.49
*(A) Wealth index*								
Poorest	45.90	44.54	54.54	58.59	**12.69**	6.45–18.92	**1.29**	1.20–1.39
Poorer	31.68	40.67	50.97	58.22	**26.54**	20.10–32.99	**1.53**	1.41–1.67
Middle	34.90	32.13	46.45	55.34	**20.44**	12.50–28.38	**1.41**	1.29–1.55
Richer	35.76	42.49	49.66	54.79	**19.03**	9.91–28.16	**1.37**	1.25–1.50
Richest	28.47	39.94	48.45	60.61	**32.15**	23.81–40.48	**1.56**	1.43–1.71
*(B) Education status*								
No education/incomplete primary	40.47	47.49	58.07	54.98	**14.52**	4.09–24.95	**1.42**	1.25–1.61
Primary/incomplete secondary	36.65	40.04	52.15	59.59	**22.94**	17.99–27.88	**1.39**	1.32–1.48
Secondary+	36.39	30.55	45.35	54.5	**18.11**	9.37–26.85	**1.48**	1.39–1.57
*(C) Occupation status*								
Unemployed	35.57	38.83	51.79	56.86	**21.28**	16.21–26.36	**1.41**	1.33–1.49
Employee: professional, managerial, clerical	23.16	30.25	43.80	61.79	**38.63**	29.49–47.77	**1.56**	1.37–1.77
Employee: others	37.38	40.28	44.36	57.31	**19.94**	8.80–31.08	**1.47**	1.29–1.68
Self-employee	38.83	43.5	51.55	58.73	**19.90**	12.58–27.22	**1.43**	1.32–1.55
*(D) Type of residence*								
Rural	38.96	40.35	51.98	56.46	**17.50**	12.35–22.64	**1.39**	1.31–1.47
Urban	32.34	38.29	48.69	58.13	**25.79**	19.89–31.69	**1.47**	1.38–1.57
*(E) Region*								
Sumatra	29.50	30.26	33.24	45.38	**15.88**	9.23–22.53	**1.32**	1.23–1.43
Java & Bali	36.83	42.03	55.59	61.87	**25.04**	19.30–30.78	**1.49**	1.39–1.60
Kalimantan	45.25	39.84	45.49	61.31	**16.06**	7.16–24.96	**1.30**	1.19–1.44
Sulawesi	30.51	41.2	51.43	46.10	**15.59**	7.34–23.83	**1.34**	1.21–1.48
Eastern Indonesia	57.45	45.68	65.36	68.47	**11.02**	1.44–20.60	**1.44**	1.3–1.60
*(F) Place of Delivery*								
Non-health facility	37.98	40.77	51.73	58.85	**20.86**	15.11–26.61	**1.34**	1.25–1.44
Government health facility	31.37	31.77	48.82	56.86	**25.49**	16.06–34.93	**1.57**	1.42–1.74
Private Facility	33.50	40.81	49.75	56.65	**23.15**	17.37–28.94	**1.45**	1.36–1.54
*(G) Birth attendant*								
None/unskilled birth attendant	37.77	39.79	53.25	58.53	**20.76**	13.78–27.74	**1.37**	1.26–1.49
Skilled birth attendant	34.45	39.19	49.83	57.18	**22.73**	17.85–27.61	**1.45**	1.38–1.52

All estimates are age-standardised to the 2000 Indonesia standard population; ^a^pORs: time trends of breastfeeding outcomes in each five years between 2002 to 2017, adjusted by maternal characteristics and birth history

Table 2 presents changes in EIB in each wave. For the overall population, point estimates for the improvement in EIB was found to be largest in 2017 (pOR = 2.79, 95% CI = 2.44–3.21), followed by 2012 (pOR = 1.92, 95% CI = 1.67–2.21) and year 2007 (pOR = 1.21, 95% CI = 1.05–1.40). These trends are found across all population groups.

**Table 2:** Fully adjusted prevalence odds ratios (pORs) and 95% CI for women who had EIB

**Variable**	**2002/2003**		**2007**		**2012**		**2017**	
	**pOR**	**95% CI**	**pOR**	**95% CI**	**pOR**	**95% CI**	**pOR**	**95% CI**
Overall	Ref		**1.21**	1.05–1.40	**1.92**	1.67–2.21	**2.79**	2.44–3.21
*(A) Wealth index*								
Poorest	Ref		1.15	0.91–1.45	**1.65**	1.32–2.06	**2.08**	1.64–2.64
Poorer	Ref		**1.62**	1.24–2.13	**2.33**	1.79–3.03	**3.69**	2.80–4.85
Middle	Ref		0.93	0.69–1.26	**1.67**	1.23–2.27	**2.55**	1.90–3.4
Richer	Ref		1.36	0.99–1.87	**1.96**	1.43–2.68	**2.54**	1.88–3.42
Richest	Ref		1.16	0.86–1.55	**2.24**	1.69–2.97	**3.50**	2.67–4.58
*(B) Education status*								
No education/incomplete primary	Ref		**1.58**	1.16–2.14	**2.28**	1.64–3.17	**2.63**	1.77–3.91
Primary/incomplete secondary	Ref		**1.20**	1.00–1.44	**1.80**	1.50–2.15	**2.66**	2.23–3.18
Secondary+	Ref		1.07	0.86–1.34	**1.96**	1.58–2.43	**2.87**	2.34–3.53
*(C) Occupation status*								
Unemployed	Ref		1.17	0.98–1.41	**1.86**	1.55–2.23	**2.7**	2.26–3.22
Employee: professional, managerial, clerical	Ref		1.08	0.66–1.76	**2.17**	1.39–3.39	**3.25**	2.14–4.94
Employee: others	Ref		1.34	0.85–2.10	**2.04**	1.34–3.10	**3.11**	2.01–4.81
*(D) Type of residence*								
Rural	Ref		1.20	1.00–1.44	**1.83**	1.52–2.19	**2.6**	2.16–3.13
Urban	Ref		1.23	0.98–1.54	**2.05**	1.65–2.55	**3.04**	2.47–3.73
*(E) Region*								
Sumatra	Ref		1.17	0.91–1.49	**1.45**	1.15–1.84	**2.33**	1.83–2.96
Java & Bali	Ref		1.17	0.94–1.45	**2.16**	1.74–2.68	**3.13**	2.53–3.87
Kalimantan	Ref		**1.39**	1.03–1.88	1.28	0.98–1.68	**2.59**	1.90–3.53
Sulawesi	Ref		**1.89**	1.39–2.57	**2.61**	1.87–3.65	**2.3**	1.67–3.17
Eastern Indonesia	Ref		0.96	0.70–1.32	**1.88**	1.36–2.61	**2.52**	1.81–3.51
*(F) Place of Delivery*								
Non-health facility	Ref		1.19	0.99–1.44	**1.79**	1.48–2.17	**2.4**	1.94–2.97
Government health facility	Ref		1.3	0.85–1.97	**2.23**	1.46–3.39	**3.58**	2.41–5.31
Private Facility	Ref		1.23	0.98–1.55	**2.02**	1.63–2.51	**2.86**	2.33–3.50
*(G) Birth attendant*								
None/unskilled birth attendant	Ref		1.15	0.93–1.43	**1.92**	1.54–2.40	**2.37**	1.82–3.10
Skilled birth attendant	Ref		**1.26**	1.06–1.50	**1.95**	1.65–2.31	**2.93**	2.50–3.45

### Exclusive breastfeeding

EB prevalence for 2002/2003, 2007, 2012, and 2017 was 39.38% (95% CI: 32.93–45.83%), 31.07% (95% CI: 26.91–35.24%), 41.34% (95% CI: 36.43–46.25%), and 51.11% (95% CI: 47.12–55.10%), respectively. Table 3 presents the trend in prevalence of EB for the entire population, as well as within population subgroups. Overall, the pORs show that EB increased between 2002/2003 and 2017 for the entire population, as well as within some subgroups. We did not find significant differences in trends across population groups due to overlapping confidence intervals.

**Table 3:** Age-standardized proportion of women who had EB at the time of interview, by select characteristics

**Variable**	**2002/2003**	**2007**	**2012**	**2017**	**Diff. between 2002/2003** **−2017**	**95% CI**	**pOR**^**a**^	**95% CI**
Overall	39.38	31.07	41.34	51.11	**11.73**	4.14–19.32	**1.21**	1.12–1.31
*(A) Wealth index*								
Poorest	42.10	39.53	43.95	58.27	**16.18**	4.97–27.39	**1.27**	1.1–1.47
Poorer	43.59	33.76	41.44	58.71	**15.12**	2.99–27.24	1.15	0.98–1.36
Middle	50.35	34.52	48.15	39.65	−10.7	−23.36-1.95	1.06	0.89–1.25
Richer	37.54	29.47	29.30	43.91	6.37	−10.58-23.32	1.09	0.9–1.33
Richest	20.32	18.44	40.89	52.95	**32.62**	19.1–46.15	**1.72**	1.42–2.09
*(B) Education status*								
No education/incomplete primary	37.80	31.9	37.52	58.13	**20.33**	4.85–35.81	1.17	0.94–1.44
Primary/incomplete secondary	43.57	36.04	41.96	51.74	8.17	−2.69-19.03	1.12	1–1.25
Secondary+	30.34	31.22	42.3	50.77	**20.43**	8.32–32.54	**1.39**	1.24–1.57
*(C) Occupation status*								
Unemployed	42.52	33.91	44.11	53.59	**11.07**	0.97–21.17	**1.15**	1.04–1.27
Employee: professional, managerial, clerical	19.75	15.55	28.74	43.31	**23.56**	10.85–36.27	**1.64**	1.34–2.01
Employee: others	49.71	27.03	37.41	41.15	−8.56	−23.68-6.56	**1.37**	1.04–1.80
Self-employed/work for family	29.67	35.41	41.25	51.85	**22.18**	9.64–34.73	**1.3**	1.10–1.53
*(D) Type of residence*								
Rural	40.07	33.18	42.92	56.34	**16.27**	7.55–24.98	**1.22**	1.10–1.34
Urban	38.32	30.59	40.75	45.79	7.46	−5.06-19.99	**1.22**	1.08–1.37
*(E) Region*								
Sumatra	39.24	33.28	34.83	51.17	**11.92**	0.62–23.23	**1.21**	1.07–1.36
Java & Bali	36.70	25.25	44.87	51.81	**15.11**	3.42–26.8	**1.26**	1.11–1.44
Kalimantan	29.37	30.03	22.22	30.78	1.41	−9.42-12.24	1.02	0.84–1.24
Sulawesi	59.22	55.66	50.86	56.49	−2.73	−15.44-9.97	1.07	0.92–1.26
Eastern Indonesia	44.7	45.59	48.96	59.08	**14.39**	2.94–25.83	**1.22**	1.01–1.46
*(F) Place of Delivery*								
Non-health facility	42.87	33.37	39.99	55.77	**12.9**	0.52–25.27	1.09	0.96–1.24
Government health facility	32.36	41.05	39.16	48.35	15.99	−1.33-33.32	**1.22**	1.01–1.48
Private Facility	34.80	25.87	43.41	50.89	**16.08**	5.88–26.29	**1.31**	1.16–1.47
*(G) Birth attendant*								
None/unskilled birth attendant	42.63	32.05	44.45	55.21	12.58	−0.89-26.06	1.10	0.94–1.28
Skilled birth attendant	34.74	30.57	40.82	50.88	**16.14**	8.7–23.59	**1.24**	1.13–1.36

All estimates are age-standardised to the 2000 Indonesia standard population; ^a^pORs: time trends of breastfeeding outcomes in each five years between 2002 to 2017, adjusted by maternal characteristics and birth history

Table 4 presents changes in EB in each wave. Our results highlight that the improvement of EB appeared to be more pronounced in 2017 (pOR = 1.65, 95% CI = 1.30–2.09) than in 2012 (p > 0.05 for coefficient in 2012). We found a statistically significant reduction in EB in 2007 (pOR = 0.77, 95% CI = 0.60–0.98). Similar patterns in change of time trend over the study period were found in all population subgroups.

**Table 4:** Fully adjusted prevalence odds ratios (pORs) and 95% CI for women who had EB

**Variable**	**2002/2003**		**2007**		**2012**		**2017**	
	**pOR**	**95% CI**	**pOR**	**95% CI**	**pOR**	**95% CI**	**pOR**	**95% CI**
Overall	Ref		**0.77**	0.60–0.98	1.11	0.87–1.42.	**1.65**	1.30–2.09
*(A) Wealth index*								
Poorest	Ref		0.98	0.66–1.45	1.08	0.72–1.60	**2.28**	1.45–3.58
Poorer	Ref		0.76	0.47–1.22	0.77	0.46–1.28	1.53	0.93–2.51
Middle	Ref		0.78	0.45–1.34	1.07	0.61–1.90	1.09	0.65–1.84
Richer	Ref		0.65	0.35–1.20	0.72	0.38–1.37	1.25	0.69–2.24
Richest	Ref		0.66	0.34–1.27	**2.70**	1.47–4.96	**3.44**	1.90–6.22
*(B) Education status*								
No education/incomplete primary	Ref		0.72	0.42–1.25	0.88	0.46–1.67	1.88	0.98–3.62
Primary/ incomplete secondary	Ref		0.76	0.55–1.06	0.95	0.67–1.34	**1.40**	1.01–1.95
Secondary+	Ref		0.83	0.55–1.26	**1.57**	1.06–2.33	**2.22**	1.52–3.24
*(C) Occupation status*								
Unemployed	Ref		0.69	0.52–0.92	0.97	0.72–1.32	**1.49**	1.11–2.00
Employee: professional, managerial, clerical	Ref		0.82	0.42–1.62	**2.21**	1.12–4.36	**3.03**	1.63–5.63
Employee: others	Ref		1.26	0.53–2.97	1.97	0.85–4.54	**2.43**	1.03–5.71
Self-employed/work for family	Ref		1.03	0.63–1.67	1.17	0.72–1.91	**2.29**	1.40–3.77
*(D) Type of residence*								
Rural	Ref		0.91	0.67–1.23	1.02	0.75–1.37	**1.85**	1.36–2.51
Urban	Ref		**0.62**	0.42–0.93	1.21	0.82–1.79	**1.55**	1.08–2.22
*(E) Region*								
Sumatra	Ref		0.78	0.54–1.14	0.87	0.61–1.24	**1.78**	1.22–2.58
Java & Bali	Ref		0.75	0.5–1.12	1.42	0.95–2.12	**1.77**	1.22–2.59
Kalimantan	Ref		0.97	0.58–1.62	0.71	0.43–1.19	1.20	0.65–2.22
Sulawesi	Ref		0.75	0.49–1.16	0.91	0.58–1.43	1.23	0.74–2.02
Eastern Indonesia	Ref		0.77	0.45–1.30	0.73	0.44–1.21	**1.80**	1.01–3.21
*(F) Place of Delivery*								
Non-health facility	Ref		0.79	0.58–1.08	0.81	0.56–1.15	**1.74**	1.16–2.64
Government health facility	Ref		1.77	0.81–3.87	1.5	0.69–3.24	**2.13**	1.01–4.52
Private Facility	Ref		**0.53**	0.35–0.82	1.18	0.8–1.74	**1.66**	1.15–2.38
*(G) Birth attendant*								
None/unskilled birth attendant	Ref		0.84	0.59–1.19	0.92	0.61–1.38	**1.71**	1.01–2.88
Skilled birth attendant	Ref		**0.68**	0.49–0.94	1.11	0.82–1.49	**1.61**	1.22–2.14

### Continued breastfeeding up to 1 year of age

CB-1 prevalence for 2002/2003, 2007, 2012, and 2017 was 83.33% (95% CI: 77.05–89.62%), 82.62% (95% CI: 79.25–86.00%), 78.90% (95% CI: 74.78–83.01%), and 77.65% (95% CI: 73.48–81.82%), respectively. Table 5 presents the trend in prevalence of EB for the entire population, as well as within population subgroups. For the entire population, there was no statistically significant improvement in EB between 2002/2003 and 2017 (p > 0.05). However, we found statistically significant decline in CB-1 among women from the second poorest wealth quintile (pOR = 0.69, 95% CI = 0.54–0.90); women in rural areas (pOR = 0.84, 95% CI = 0.72–0.98); and women in Kalimantan (pOR = 0.76, 95% CI = 0.60–0.97) and Sulawesi (pOR = 0.80, 95% CI = 0.65–0.98).

**Table 5:** Age-standardised proportion of women who had CB-1, by select characteristics

**Variable**	**2002/2003**	**2007**	**2012**	**2017**	**Diff. between 2002/2003–2017**	**95% CI**	**pOR**^**a**^	**95% CI**
Overall	83.33	82.62	78.9	77.65	−5.69	−13.24-1.86	0.93	0.84–1.04
*(A) Wealth index*								
Poorest	84.65	83.78	87.11	89.71	5.05	−2.92-13.02	0.98	0.80–1.20
Poorer	91.03	87.14	79.09	86.25	−4.78	−13.91-4.36	**0.69**	0.54–0.90
Middle	88.71	86.27	79.65	71.93	**−16.78**	−30.52--3.04	0.93	0.75–1.15
Richer	76.44	69.51	77.73	64.42	−12.01	−26.70-2.67	1.02	0.84–1.23
Richest	77.63	78.56	74.82	72.06	−5.57	−16.30-5.16	0.93	0.75–1.16
*(B) Education status*								
No education/incomplete primary	91.58	82.92	85.65	88.2	−3.38	−10.22-3.46	0.80	0.60–1.08
Primary/incomplete secondary	89.81	86.85	81.85	82.37	**−7.44**	−14.22--0.65	0.90	0.77–1.05
Secondary+	63.69	77.45	74.12	71.37	7.68	−2.06-17.42	0.97	0.83–1.13
*(C) Occupation status*								
Unemployed	86.36	87.83	82.18	80.39	−5.97	−14.72-2.79	0.90	0.77–1.06
Employee: professional, managerial, clerical	76.19	70.84	50.1	46.54	**−29.65**	−45.46--13.84	0.87	0.68–1.11
Employee: others	71.48	64.41	68.69	70.63	−0.85	−17.37-15.66	1.03	0.78–1.37
Self-employed/work for family	84.94	82.49	83.75	76.17	−8.77	−20.94-3.40	1.03	0.84–1.28
*(D) Type of residence*								
Rural	88.37	86.11	84.61	82.96	−5.41	−11.83-1.00	**0.84**	0.72–0.98
Urban	76.92	78.78	73.18	68.14	−8.78	−21.57-4.00	1.00	0.87–1.16
*(E) Region*								
Sumatra	87.01	79.75	83.44	80.77	−6.24	−13.97-1.48	0.95	0.81–1.10
Java & Bali	81.6	84.91	79.59	77.36	−4.25	−16.03-7.53	0.99	0.83–1.18
Kalimantan	80.11	82.81	76.91	67.96	−12.15	−29.44-5.15	**0.76**	0.60–0.97
Sulawesi	89.64	78.31	74.29	76.6	**−13.04**	−22.03--4.06	**0.80**	0.65–0.98
Eastern Indonesia	83.18	75.79	82.2	79.51	−3.67	−14.69-7.35	0.84	0.67–1.06
*(F) Place of Delivery*								
Non-health facility	90.42	86.09	85.71	83.08	**−7.34**	−13.81--0.87	**0.81**	0.68–0.96
Government health facility	80.34	83.36	73.42	79.2	−1.14	−14.14-11.86	0.81	0.65–1.01
Private Facility	70.55	78.8	77.26	74.63	4.08	−9.79-17.95	1.07	0.92–1.26
*(G) Birth attendant*								
None/unskilled birth attendant	90.44	85.28	85.17	81.33	−9.11	−18.31-0.09	0.81	0.66–1.00
Skilled birth attendant	77.81	80.91	76.42	77.17	−0.64	−10.78-9.49	0.97	0.86–1.09

All estimates are age-standardised to the 2000 Indonesia standard population; ^a^pORs: time trends of breastfeeding outcomes in each five years between 2002 to 2017, adjusted by maternal characteristics and birth history

Table 6 presents changes in CB-1 in each wave. Based on socioeconomic characteristics, the decline of CB-1 was found to be statistically significant among women from the second poorest wealth quintile in 2012 (pOR = 0.27 in 2012, pOR = 0.30 in 2017, *p* < 0.05). The decline in CB-1 for women in professional sectors (pOR = 0.42, 95% CI = 0.18–0.94) and women from Kalimantan (pOR = 0.49, 95% CI = 0.25–0.93) was large in 2012 compared to 2002/2003; however, this observation did not remain significant in 2017 (p > 0.05). The decline in CB-1 for women from Sulawesi was found to be statistically significant in 2007 (pOR = 0.40, 95% CI = 0.20–0.78), 2012 (pOR = 0.36, 95% CI = 0.19–0.71), and 2017 (pOR = 0.45, 95% CI = 0.22–0.92).

**Table 6:** Fully adjusted prevalence odds ratios (pORs) and 95% CI for women who had CB-1

**Variable**	**2002/2003**		**2007**		**2012**		**2017**	
	**pOR**	**95% CI**	**pOR**	**95% CI**	**pOR**	**95% CI**	**pOR**	**95% CI**
Overall	Ref		0.77	0.55–1.09	0.77	0.54–1.09	0.78	0.54–1.11
*(A) Wealth index*								
Poorest	Ref		0.73	0.39–1.35	1.03	0.57–1.87	0.85	0.44–1.66
Poorer	Ref		0.47	0.21–1.10	**0.27**	0.12–0.63	**0.30**	0.13–0.70
Middle	Ref		0.64	0.32–1.29	0.83	0.38–1.83	0.68	0.34–1.38
Richer	Ref		1.05	0.53–2.09	0.81	0.42–1.56	1.13	0.60–2.13
Richest	Ref		0.89	0.46–1.74	0.88	0.42–1.82	0.8	0.40–1.61
*(B) Education status*								
No education/incomplete primary	Ref		0.5	0.23–1.12	0.63	0.28–1.41	0.46	0.17–1.20
Primary/incomplete secondary	Ref		0.72	0.43–1.20	0.71	0.42–1.19	0.69	0.41–1.18
Secondary+	Ref		0.91	0.53–1.57	0.86	0.49–1.49	0.9	0.54–1.53
*(C) Occupation status*								
Unemployed	Ref		0.78	0.48–1.27	0.73	0.44–1.21	0.72	0.43–1.22
Employee: professional, managerial, clerical	Ref		0.53	0.20–1.42	**0.42**	0.18–0.94	0.51	0.24–1.09
Employee: others	Ref		0.67	0.27–1.67	0.97	0.40–2.36	0.91	0.37–2.24
Self-employed/work for family	Ref		0.96	0.52–1.78	1.04	0.53–2.04	1.09	0.56–2.10
*(D) Type of residence*								
Rural	Ref		0.71	0.44–1.14	0.68	0.41–1.13	**0.55**	0.33–0.92
Urban	Ref		0.79	0.48–1.30	0.84	0.51–1.36	0.96	0.59–1.55
*(E) Region*								
Sumatra	Ref		0.64	0.38–1.08	0.75	0.45–1.24	0.77	0.46–1.30
Java & Bali	Ref		1.00	0.57–1.77	0.99	0.55–1.77	0.97	0.55–1.70
Kalimantan	Ref		0.81	0.37–1.77	**0.49**	0.25–0.93	0.48	0.22–1.06
Sulawesi	Ref		**0.40**	0.20–0.78	**0.36**	0.19–0.71	**0.45**	0.22–0.92
Eastern Indonesia	Ref		0.64	0.27–1.48	0.56	0.24–1.29	0.54	0.23–1.25
*(F) Place of Delivery*								
Non-health facility	Ref		0.63	0.39–1.02	0.65	0.39–1.08	**0.48**	0.27–0.86
Government health facility	Ref		0.69	0.29–1.63	0.54	0.22–1.36	0.49	0.21–1.15
Private Facility	Ref		0.99	0.57–1.74	1.04	0.60–1.81	1.23	0.72–2.08
*(G) Birth attendant*								
None/unskilled birth attendant	Ref		**0.52**	0.30–0.90	0.67	0.36–1.24	**0.42**	0.21–0.85
Skilled birth attendant	Ref		0.93	0.61–1.42	0.85	0.55–1.30	0.91	0.61–1.36

## Discussion

### Principal findings

This study is the first investigation into the trends and patterns of breastfeeding practices across differing subpopulations between 2002/2003 and 2017 in Indonesia using four nationally representative surveys. It is also the first study to examine the pattern of EB rates after the introduction of legislations to promote breastfeeding in Indonesia between 2003 and 2012, as described above. Our findings indicate that prevalence of both EIB and EB has improved in Indonesia from 2002/2003 to 2017, coinciding with a higher rate of health facility deliveries over this period [39]. However, there remains a high proportion of women who reported not undertaking EIB (43.7%) or EB (48.9%) in 2017. In contrast, we did not find a statistically significant decline in CB-1 over time for the overall population, except for women who: were from the second-lowest wealth quintile; were rural dwelling; delivered in non-health facility; and lived in Kalimantan and Sulawesi.

### Previous studies

Our findings are broadly consistent with evidence from other LMICs which show that breastfeeding practice, especially EIB and EB, have been improving in recent years [40, 41]. Analysis from Chai et al. [42] suggested the increased in EB rates in most LMICs was affected by the decline in suboptimal feeding practices, such as the consumption of infant formula, water, or juices. While this study observed improved rates of EIB in Indonesia, during the period from 2002 to 2017, approximately 40% of women were not engaging in EIB. Suboptimal improvement in EIB might be affected by lower engagement with the BFHI in Indonesia. Based on a 2015 World Breastfeeding Trends Initiative (WBTi) report, many hospitals in Indonesia have not yet fully implemented the Ten Steps of Successful Breastfeeding (the Ten Steps) as part of BFHI [26]. A UK study found that the implementation of BFHI in hospitals effectively increased the prevalence of EIB [43]. In Indonesia of the 685 hospitals offering maternity services, only 55 hospitals (8%) had implemented BFHI [26]. In addition, the BFHI program implemented in Indonesia does not fully adopt the WHO recommendation, as some procedures in the Ten Steps were omitted [26].

Our findings indicate that the improvement in EIB and EB tended to be larger for women from the highest wealth quintile, as well as those who are more highly educated in recent years. For EIB, the increase was larger for more highly educated women in 2017 and 2012, compared with 2002/2003. The improvement in EB was more significant in 2017 and 2012 for women from the highest wealth quintile, as well as those who completed secondary education. This finding is consistent with a meta-analysis by Victora et al. [8], which found that the increase in EB rate was steeper for women from wealthier households, while the poorest followed the general trend. A study in Nepal also found that the odds of EIB for women with primary and secondary education increased in recent years [44]. The rise in EIB and EB rates in recent years may be evidence that the breastfeeding legislations introduced by the GoI since 2003 have promoted the rise in optimal breastfeeding practices across the population.

Although the improvement of EB practice in Indonesia is marked, it remains that almost half of infants in Indonesia were not exclusively breastfed at the time of the 2017 IDHS. There were also persistent disparities of EB based on occupational, and geographical groups. Our findings also demonstrate that the EB rate among women working in professional sectors remained low in 2002 to 2017. This result is consistent with studies carried out in Indonesia, which have reported lower odds of EB for women employed in both the private or public sectors [28, 45]. Indonesia has not yet ratified the Maternity Protection Recommendation (R191) of the International Labour Organization (ILO) to provide at least 18 weeks of maternity leave [46]. An analysis of data from 38 LMIC in 2018 indicates that extending the duration of maternity leave is associated with a 5.9 percentage point increase in EB rates and an increase in breastfeeding duration of 2 months [42]. Spagnoletti et al. [15] described employer inflexibility in Indonesia concerning the commencement of paid maternity leave, and the relatively short duration of paid maternity leave postpartum, which was typically only 6 weeks. Their analysis highlighted that among women with access to paid maternity leave who return to work, the majority of EB duration (6 months, as recommended by the WHO) would be undertaken in the workplace. However, many working women in Indonesia do not have access to ideal facilities to express and store breastmilk in the workplace, such as a private room and refrigeration. Even though legislation requires employers to provide breastfeeding breaks and lactation rooms, studies have found its implementation to be uneven. Basrowi et al. [17] (n = 186) and Spagnoletti et al. [15, 18] (n = 20) found that many women did not have access to an appropriate lactation room in their workplaces. A recent study of Cambodian factory workers (n = 109) found women gave infant formula to their children, rather than expressed breastmilk due to a mistrust of refrigeration for breastmilk storage at workplaces [47]. Similar issues may influence the infant feeding decisions of Indonesian women, many of whom are factory workers [48, 49].

The promotion of infant formula also discourages EB practice in Indonesia [24]. The ease of infant formula access; aggressive marketing in retail outlets and the media; continued provision of formula samples and feeding supplies by health professionals; and lack of comprehensive understandings of optimal infant nutrition and feeding practices, are some of the reasons that families choose to use infant formula [13, 24]. Violations of the Code by infant formula companies include incentivizing the provision of infant formula and feeding supplies by health professionals and retail outlets, and producing misleading advertisements and product labelling [25]. In the US, the government formed the National Alliance for Breastfeeding Advocacy (NABA) to monitor compliance with the Code and establish a task force to identify hospitals which have collaborated with infant formula companies [50]. In Indonesia the 2012 Regulation stipulates large fines for health services, and fines and suspension of license to practice for health professionals found to be promoting infant formula, however the mechanism for monitoring this is unclear [18]. In Demak, Central Java, a multilevel EB promotion and monitoring intervention has successfully increased EB duration [30, 31]. Such an approach which engages communities, families, and individuals, might be suited to other parts of rural Indonesia.

This study found greater decline in CB-1 prevalence – both for women from poorer wealth quintiles, and for women from rural areas – in 2017 compared to 2002/2003. A similar pattern was seen in a trend study in LMIC, which found that CB-1 decreased globally due to declining breastfeeding among women from the poorest households [8]. Another study revealed that women from lower-income households were found to have less family support, less working flexibility, and face greater barriers to accessing assistance for breastfeeding problems [51]. These findings indicate that health promotion efforts to support prolonged breastfeeding practice should be targeted at poorer and rural communities.

Finally, another important finding from this study is the regional differences in breastfeeding across Indonesia. Women from Java and Bali reported improved EB and EIB practices in the last 15 years. Women in Java and Bali also had higher odds of EIB, EB, and CB-1 compared to women in Sumatra. The differences in breastfeeding improvement are likely due to Java and Bali having better infrastructure and greater density of health services, compared to other regions [39, 52]. Lo Bue and Priebe [28] posit that the availability and quality of health services, along with growth in demand from women could increase the EB rate in Indonesia. This study also found that women from Sumatra and Sulawesi had lower EIB rates in 2017 compared to other regions. Some local studies indicate that limited knowledge of EIB for both health workers and mothers, and the lack of local government guidelines to support EIB, cause suboptimal implementation of EIB in both regions [53, 54].

### Limitations

Our study had several limitations. The survey data use a cross-sectional design, limiting inferences on causation. There is also the possibility of response bias due to the recall method used to assess EIB and EB. Munos et al. [55] emphasised that women’s recall of interventions that occur immediately after a delivery, including EIB, have low level of validity. On the other hand, the EB variable was assessed using 24-h recall, which could produce overestimates in EB proportion as it does not capture the usual infant feeding pattern [56]. The sample size of women which had children 12–15 months were smaller than women who had children less than 6 months, which resulted in lower statistical power to detect changes over time in CB-1 [27]. The high proportion of continued breastfeeding also does not reflect optimal breastfeeding practices, where women had EIB, EB as the survey used a cross-sectional design for which detailed information regarding history of optimal breastfeeding practices is unavailable [4]. Our findings revealed that large disparities remain in breastfeeding practices across population groups, emphasising that further studies are warranted to investigate the effect of individual and population wide interventions, and their impact on health inequalities in Indonesia.

### Clinical and policy implications and future research

While the GoI has introduced several pieces of legislation since 2002 to promote breastfeeding and EB in Indonesia, their enforcement and implementation need improvement. There is a need to improve breastfeeding promotion in maternal health care facilities and hospitals. For the minority of hospitals that are already BFHI accredited, this would involve the full adoption of BFHI and the Ten Steps. Moreover, as BFHI accredited facilities comprise fewer than 1 in 10 Indonesian hospitals, barriers to scaling up BFHI accreditation in Indonesia should explored and addressed. Non-hospital maternal health care facilities also play an important role in educating expectant mothers and assisting births in Indonesia, and the expansion of the BFHI to include such facilities should also be considered.

Infant formula promotion and provision is a major barrier to breastfeeding promotion in Indonesia. Addressing it would require the full adoption and enforcement of the Code, expansion of the Code to include midwives and other community health workers, and high profile prosecution when the Code is breached. There is also a need for enhanced breastfeeding education to expecting parents, families, and community health workers to develop greater community health literacy of infant nutrition and optimal feeding practices.

This study has also highlighted that CB-1 is declining among some Indonesian women. There is a need for further research to explore the reasons for this decline and identify appropriate interventions.

A larger number of Indonesian women are completing high school and pursuing university education, widening their employment opportunities. While there is legislation in provide maternity protections to some working women, this could be strengthened. At the policy level, Indonesia has attempted to support women’s continued engagement in the workforce during their childbearing years and in conjunction with breastfeeding. Yet the implementation of the 2009 Health Law and 2012 Health Regulation in workplaces has been uneven, with no evidence of monitoring. Workplace implementation of breastfeeding policy and the provision of facilities is an area for future research and policy reform. In particular there is a need to understand the potential constraints that employers face in transforming the legislation into practice. The GoI should also revisit the Labor Law of 2003 to better support working women to exclusively breastfeed. The availability of part time work or job-sharing options, which may support women to breastfeed as they transition from maternity leave to the workforce [15], is not commonplace in Indonesia [57]. A possible approach is by expanding the paid maternity leave to a minimum of 18 weeks, as recommended by ILO. However, more research on how this policy can be effectively implemented in Indonesia is required.

## Conclusion

While the findings from this study indicate overall improvement in breastfeeding practices in Indonesia from 2002 to 2017, coinciding with the introduction of breastfeeding promotions legislation by the GoI, large disparities exist. Consequently, there is a need for further exploration of the factors contributing to the decline in breastfeeding practices for certain subgroups of women, particularly women working in the professional sectors, and women living outside of Java and Bali. Targeted policies and strategies will be necessary to improve breastfeeding practices among all groups of Indonesia women to reduce disparities.

## Figures and Tables

**Fig. 1:**
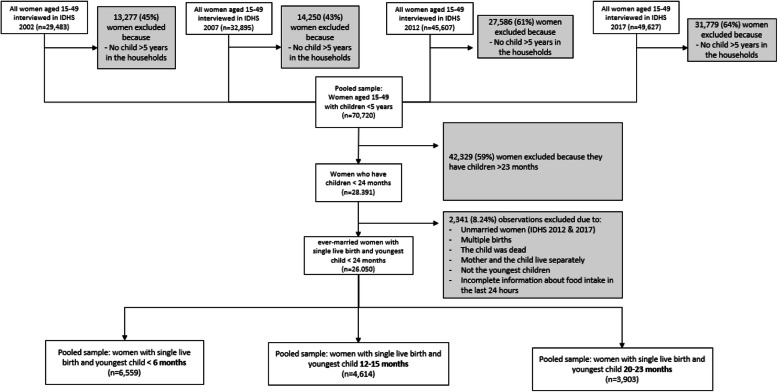
Sample flow-chart

## Data Availability

This study used datasets available from USAID’s DHS Program and can be accessed at https://dhsprogram.com/data/available-datasets.cfm

## Appendix 2

**Table 7:** List of variables

Variable	Type	Measurement	Source of measurement^a^
**Dependent variable**			
Early initiation of breastfeeding (Child aged 0–23 months)	Binary	0. No 1. Yes	Women’s questionnaire: q441: How long after birth did you first put (NAME) to the breast?
Exclusive breastfeeding (Child aged 0–5 months)	Binary	0. No 1. Yes	Women’s questionnaire: q445: Are you still breastfeeding (NAME)? q492: Now I would like to ask you about liquids (NAME) **drank** over the last seven days, including yesterday. In total, how many times yesterday during the day or at night did (NAME FROM Q. 491) drink (ITEM)? *ITEM LIST: plain water, commercially produced infant formula, any other milk such as condensed sweetened milk, powdered, or fresh animal milk, fruit juice, any other liquids such as sugar water, tea, coffee, carbonated drinks, or soup broth* q493. Now I would like to ask you about the types of foods (NAME) **ate** over the last seven days, including yesterday. In total, how many times yesterday during the day or at night did (NAME FROM Q. 491) drink (ITEM)? *ITEM LIST: any food made from grains,* e.g.*, maize, rice, sago or other local grains, pumpkin, sweet potatoes or yams or carrots, any other foods made from roots or tubers,* e.g.*, potatoes, white sweet potatoes, cassava, or other local roots/tubers, any green leafy vegetables, such as spinach, cassava leaves, mango, papaya, durian, jackfruit or other yellow and red fruits, any other fruits and vegetables,* e.g.*, bananas, apples, green beans, peas, avocados, tomatoes, meat, poultry, fish, shellfish, or eggs, any food made from legumes,* e.g.*, tofu, tempeh, lentils, beans, soybeans, pulses, or peanuts, cheese or yoghurt, any food made of oil, fat or butter*
Cont. breastfeeding at 1 year (child aged 12–15 months)	Binary	0. No 1. Yes	Women’s questionnaire: q445: Are you still breastfeeding (NAME)?
**Independent variable**			
Region	Categorical nominal	1. Sumatra 2. Java & Bali 3. Kalimantan 4. Sulawesi 5. Eastern Indonesia	Women’s questionnaire: q1: Province
Type of residence	Categorical nominal	0. Rural 1. Urban	Women’s questionnaire: q5: Urban/rural
Maternal education	Categorical ordinal	1. No education/incomplete primary 2. Primary/ incomplete secondary 3. Secondary+	Women’s questionnaire: q108: What is the highest level of school you attended: primary, junior high, senior high, academy or university?
Maternal occupation	Categorical nominal	0. Unemployed 1. Employee: professional 2. Employee: others 3. Self-employed/work for family	q710: What is your (most recent) occupation, that is, what kind of work (do/did) you mainly do? q713: Do you do this work for a member of your family, for someone else, or are you self-employed?
Wealth	Categorical ordinal	1. Poorest 2. Poorer 3. Middle 4. Richer 5. Richest	https://www.dhsprogram.com/topics/wealth-index/Wealth-Index-Construction.cfm
Place of delivery	Categorical nominal	0. Non-health facility 1. Government health facility 2. Private health facility	q427: Where did you give birth to (name)?
Birth assistance	Categorical nominal	0. Non-skilled birth attendant 1. Skilled birth attendant	q426: Who assisted with delivery of (name)?

^a^Variable names are derived from IDHS 2002/2003. In the IDHS, 2007, 2012 and 2017, the variable names differ, however the content (i.e. what the variable actually represents) remains the same

## Appendix 3

**Table 8:** Sample characteristics IDHS 2002/2003–2017

Variable	Women with children aged 0–23 months (*n* = 26,050)				
	2002/2003 (%)	2007 (%)	2012 (%)	2017 (%)	Pooled data 2002/2003–2017 (%)
*External environment*					
Region					
Sumatra	22.99	22.65	22.64	23.3	22.89
Java & Bali	56.31	54.79	55.92	56.47	55.86
Kalimantan	6.91	6.35	6.53	5.9	6.41
Sulawesi	8.92	8.34	7.76	7.32	8.06
Eastern Indonesia	4.87	7.86	7.14	7.01	6.78
Type of residence					
Rural	52.98	58.19	50.44	51.11	53.14
Urban	47.02	41.81	49.56	48.89	46.86
*Maternal characteristics*					
Education status					
No education/incomplete primary	17.2	12.62	8.71	6.03	10.93
Primary/incomplete secondary	54.54	54.97	50.26	46.2	51.37
Secondary+	28.26	32.42	41.02	47.77	37.7
Occupation status					
Unemployed	66.29	58.32	54.81	56.64	58.76
Employee: professional, managerial, clerical	4.74	5.86	9.64	11.3	8.01
Employee: others	9.31	11.33	11.53	11.04	10.85
Self-employed/work for family	19.66	24.49	24.03	21.01	22.38
Wealth					
Poorest	22.19	21.04	20	19.38	20.6
Poorer	18.95	18.48	21.41	20.25	19.82
Middle	19.76	20.83	19.56	19.96	20.03
Richer	20.03	20.32	20.34	21.19	20.48
Richest	19.07	19.33	18.68	19.22	19.07

## Abbreviations

IDHS: Indonesia Demographic and Health Survey
EIB: Early initiation of breastfeeding
EB: Exclusive breastfeeding
CB-1: Continued breastfeeding at first year
SDG: Sustainable development goals
WHO: World Health Organization
LMIC: Low-middle-income country
*Riskesdas*: Basic health survey (*Riset kesehatan dasar*)
GoI: Government of Indonesia
the Code: International Code of Marketing of Breastmilk substitutes
BFHI: Baby-friendly hospital initiatives
PCA: Principal component analysis
PSU: Primary sampling unit
pOR: Prevalence odds ratio
CI: Confidence interval
WBTi: World Breastfeeding Trends Initiative
the Ten Steps: the Ten Steps of Successful Breastfeeding
ILO: International Labour Organization
NABA: National Alliance for Breastfeeding Advocacy

## Footnotes

1 The IDHS has been carried out in all provinces in Indonesia, with the exception of Nanggroe Aceh Darussalam, Maluku, North Maluku, and Papua in 2002/2003 due to security reasons [32].
